# Lupin protein isolate versus casein modifies cholesterol excretion and mRNA expression of intestinal sterol transporters in a pig model

**DOI:** 10.1186/1743-7075-11-9

**Published:** 2014-02-03

**Authors:** Juliane Radtke, Stefanie Geissler, Alexandra Schutkowski, Corinna Brandsch, Holger Kluge, Marcello M Duranti, Sylvia Keller, Gerhard Jahreis, Frank Hirche, Gabriele I Stangl

**Affiliations:** 1 Institute of Agricultural and Nutritional Sciences, Martin Luther University Halle-Wittenberg, Von-Danckelmann-Platz 2, 06120 Halle (Saale), Germany; 2 Department of Food, Environmental and Nutritional Sciences, Università degli Studi di Milano, Via Giovanni Celoria 2, 20133 Milano, Italy; 3 Department of Nutritional Physiology, Institute of Nutrition, Friedrich Schiller University Jena, Dornburger Str. 24, 07743 Jena, Germany

**Keywords:** Lupin protein isolate, Faecal cholesterol output, Intestinal sterol transporters, Cholesterol-uptake, Pigs, Caco-2 cells

## Abstract

**Background:**

Lupin proteins exert hypocholesterolemic effects in man and animals, although the underlying mechanism remains uncertain. Herein we investigated whether lupin proteins compared to casein modulate sterol excretion and mRNA expression of intestinal sterol transporters by use of pigs as an animal model with similar lipid metabolism as humans, and cellular cholesterol-uptake by Caco-2 cells.

**Methods:**

Two groups of pigs were fed cholesterol-containing diets with either 230 g/kg of lupin protein isolate from *L. angustifolius* or 230 g/kg casein, for 4 weeks. Faeces were collected quantitatively over a 5 d period for analysis of neutral sterols and bile acids by gas chromatographically methods. The mRNA abundances of intestinal lipid transporters were analysed by real-time RT-PCR. Cholesterol-uptake studies were performed with Caco-2 cells that were incubated with lupin conglutin γ, phytate, ezetimibe or albumin in the presence of labelled [4-^14^C]-cholesterol.

**Results:**

Pigs fed the lupin protein isolate revealed lower cholesterol concentrations in total plasma, LDL and HDL than pigs fed casein (*P* < 0.05). Analysis of faeces revealed a higher output of cholesterol in pigs that were fed lupin protein isolate compared to pigs that received casein (+57.1%; *P* < 0.05). Relative mRNA concentrations of intestinal sterol transporters involved in cholesterol absorption (Niemann-Pick C1-like 1, scavenger receptor class B, type 1) were lower in pigs fed lupin protein isolate than in those who received casein (*P* < 0.05). *In vitro* data showed that phytate was capable of reducing the uptake of labelled [4-^14^C]-cholesterol into the Caco-2 cells to the same extend as ezetimibe when compared to control (−20.5% vs. −21.1%; *P* < 0.05).

**Conclusions:**

Data reveal that the cholesterol-lowering effect of lupin protein isolate is attributable to an increased faecal output of cholesterol and a reduced intestinal uptake of cholesterol. The findings indicate phytate as a possible biofunctional ingredient of lupin protein isolate.

## Background

Diets that contain lupin proteins have repeatedly been shown to lower plasma cholesterol concentrations in hypercholesterolemic rats [1-3] and humans [4] compared to casein. Those studies demonstrated higher protein concentration and activity of hepatic low density lipoprotein (LDL) receptor, and a transcriptional up-regulation of hepatic cholesterol 7α-hydroxylase (CYP7A1), a key enzyme involved in the synthesis of bile acids, in response to lupin proteins [1-3]. However, the mechanism by which ingested lupin proteins can alter hepatic LDL receptor and hepatic enzyme expression remains uncertain. It may be argued whether the observed alterations in liver lipid metabolism can be attributed to intact lupin polypeptides that enter the organism. However, it appears more plausible that lupin proteins and/or lupin protein-associated compounds, respectively, exert their physiological effects in the intestine which in turn indirectly alter the hepatic protein and mRNA concentrations of LDL receptor and CYP7A1. As a matter of facts, recent data demonstrate that pigs fed a diet with blue lupin seeds had lower plasma cholesterol concentrations and a higher faecal output of neutral sterols than casein fed pigs [5]. Those authors assumed that the hypocholesterolemic effect of the blue lupin seeds, which were added to the diet as substitute for corn starch, soybean oil and casein, was attributable to impaired intestinal cholesterol absorption. However, whole lupin seeds contain not only proteins but also fibres that may have caused the increased sterol output in that study. Whether isolated lupin proteins themselves could contribute to the increased cholesterol excretion has not been demonstrated yet, though there is evidence suggesting that proteins cause these kind of effects. Ovariectomised cynomolgus monkeys that were fed isolated soybean proteins for 20 weeks absorbed less cholesterol in the intestine than monkeys which received casein alone or supplemented with soy isoflavones [6]. In that study soybean proteins revealed a specific impact on cholesterol output, whilst the excretion of bile acids was not influenced in response to soybean proteins. In view of the considerable genetic similarity between soy and lupin, it is most likely that the cholesterol-lowering effect of lupin proteins is also caused by an increased sterol output.

The current study aimed to examine the effect of lupin protein isolate compared to casein on faecal output of neutral and acidic sterols, and on the expression of intestinal sterol transporters. Pigs were used as an animal model because morphology and physiology of the gastrointestinal system, the ingesta transit times, the digestive efficiencies and the response to hyperlipidemic diets are comparable to those of humans [7]. Since lupin proteins have been shown to be most effective in lowering plasma cholesterol concentrations when administered together with a hypercholesterolemic diet [2], all pigs received a cholesterol-containing diet. Besides parameters that enable conclusions on intestinal sterol absorption, the bioavailability of minerals and fat-soluble vitamins were analysed.

Since *in vitro* experiments with a buckwheat protein product [8] and soy protein hydrolysate [9] showed reduced cholesterol-uptake by Caco-2 cells compared to control, similar experiments were conducted to investigate which component of the lupin protein isolate is responsible for the lipid lowering effect. To this aim, Caco-2 cells were incubated with pure conglutin γ, a candidate cholesterol-lowering lupin seed protein in force of its ability to reduce the LDL receptor activity in HepG2 cells [1], and phytate, which naturally occurs in isolates of plant proteins like lupin proteins. Incubations were performed in the presence of radiolabelled [4-^14^C]-cholesterol to quantify the cholesterol-uptake.

## Methods

### Animals, diet and design

Twenty-four 12 weeks old female crossbred pigs [(German Landrace × Large White) x Pietrain] with an initial body weight of 34.9 ± 1.9 kg (mean ± SD) were randomly assigned to 2 groups of 12 pigs each. The animals received a hypercholesterolemic basal diet, one group with 230 g/kg lupin protein isolate from *L. angustifolius* and the other group (control) with 230 g/kg casein (Meggle, Wasserburg, Germany). The diets were fed for 4 weeks. Besides the experimental proteins, the hypercholesterolemic basal diet contained (in g/kg diet) wheat (400), corn starch (casein diet: 197; lupin protein diet: 192), coconut fat (120), cholesterol (20), vitamin and mineral mixture (20) and monocalcium phosphate (13). The lupin protein diet was supplemented additionally with 5 g/kg of DL-methionine at the expense of corn starch to meet the requirement for methionine for growing pigs [10]. Minerals and vitamins were added according to the recommendations of the NRC [10] as precast mix (Mineral feed, Basu, Bad Sulza, Germany). The diets were calculated on the basis of GfE recommendations [11] to contain 15 MJ/kg. The pigs were individually kept in pens in an environmentally controlled facility with a temperature of 20°C, relative humidity between 55-60%, and light from 06:00 am to 06:00 pm. All pigs had free access to food. Water was available *ad libitum* from a nipple drinker system during the whole experiment. The pigs were weighed once a week. During the last 5 days of the experiment, six pigs of each group were kept in metabolism cages which allowed a quantitative collection of faeces. The experimental procedure was performed according to the established guidelines for care and handling of laboratory animals and was approved by the council of Saxony-Anhalt, Germany (No. 42502-2-1040MLU).

### Preparation and characterisation of the experimental proteins

Casein that was used as control protein was not further processed. Defatted total protein isolate of *L. angustifolius* was provided by the Fraunhofer Institute (IVV, Freising, Germany). The crude protein content of the diet proteins was determined by official methods [12], and was 948 g/kg dry matter (DM) for casein and 915 g/kg DM for lupin protein isolate. The amino acid composition of the experimental proteins is shown in Additional file 1. Casein contained (in g/kg DM) 0.58 calcium, 2.7 phosphorus, 0.10 magnesium, and 0.056 zinc. The lupin protein isolate contained (in g/kg DM) 1.02 calcium, 9.41 phosphorus, 0.59 magnesium, and 0.032 zinc. The calculated amount of phytic acid based on the analysed phytate phosphate in the lupin protein isolate was 25.4 g/kg DM. No detectable phytate phosphate was found in casein.

Lupin conglutin γ was isolated from lupin seed flour according to Lovati et al. [13]. The procedure consisted of various chromatographic steps, including metal affinity chromatography on NiNTA-agarose. The homogeneity of purified conglutin γ was assessed by SDS-PAGE [13].

### Amino acid analysis of the experimental proteins

Samples were oxidised and then hydrolysed with 6 M HCl [12] to determine the amino acid concentrations. Separation and quantification of the amino acids were performed by ion-exchange chromatography following post-column derivatisation in an amino acid analyzer (Biotronic LC 3000; Eppendorf, Hamburg, Germany). After digesting the diets with barium hydroxide [14] tryptophan was quantified by reversed-phase HPLC [15].

### Analysis of phytate phosphate and phytic acid of the experimental proteins

The concentration of phytate phosphate in the dietary proteins was analysed by a method of Harland and Oberleas [16]. Briefly, phytic acid was extracted from 2 g protein with 40 ml 2.4% HCl. Afterwards the samples were filtered and immobilised onto an anion exchange column (AG1-X4, 100–200 mesh, chloride form, Bio-Rad Laboratories, Hercules, California, USA). After washings the retained inositol phosphates were eluted from the column with 0.7 M NaCl. A 50 μl aliquot of the eluent was hydrolysed with 50 μl of a 5 M HClO_4_ and 1 M H_2_SO_4_ mixture, and was ashed at 250°C. The inorganic phosphate concentration of the samples was quantified colourimetrically by use of standards. The phytic acid content of the sample was calculated to be 28.2% × phosphorus [16].

### Sample collection

Blood samples that were taken from each animal at the beginning and at the end of the experiment were collected in heparinised tubes and centrifuged at 4°C and 1,100 g for 10 min to obtain plasma. Faeces were collected twice a day over a period of 5 days. Pooled faeces from each pig were frozen at −20°C pending analysis. At the end of the experimental period, the pigs were anaesthetised and killed by exsanguination 12 h after their last meal. The liver was harvested and weighed. Thirty cm of small intestine (10 cm before and 20 cm behind the *papilla duodeni major*) were removed, rinsed with physiological NaCl solution and cut lengthwise. The intestinal mucosa was scraped and mixed. Samples of liver and intestinal mucosa were snap-frozen in liquid nitrogen and stored at −80°C pending further analysis.

### Lipoprotein isolation and lipid analysis

Plasma lipoproteins were separated according to their density (very low density lipoprotein, VLDL, ρ < 1.006 g/ml; LDL, 1.006 g/ml < ρ < 1.040 g/ml; high density lipoprotein, HDL, 1.063 g/ml < ρ < 1.21 g/ml) by step wise ultracentrifugation. Lipids from liver and freeze dried faeces were extracted with a mixture of n-hexane and isopropanol (3:2, v/v) according to the method from Hara & Radin [17] modified by Eder & Kirchgessner [18]. To measure lipid concentrations of liver and faeces, aliquots of the lipid extracts were dried and residues dissolved in a mixture of Triton X-100 and chloroform (1:1, w/w). The cholesterol and triglyceride concentrations of plasma, plasma lipoproteins, liver and faeces were determined using enzymatic reagent kits (Diagnostic Systems, Holzheim, Germany).

### Analysis of faecal neutral sterols and bile acids by GC-FID and GC-MS

Lyophilised aliquots of homogenised faeces were analysed for neutral sterols (cholesterol, coprostanol, cholestanol, coprostanone and cholestanone) and for bile acids (iso-lithocholic acid, lithocholic acid, chenodeoxycholic acid, hyodeoxycholic acid, hyocholic acid) according to gas chromatographically methods, which have been previously described [19,20]. In brief, 50 mg faeces were provided with 5α-cholestane (Sigma-Aldrich, Taufkirchen, Germany) as internal standard and underwent a mild alkaline hydrolysis with freshly prepared 1 M ethanolic NaOH. Free sterols were extracted with cyclohexane, extracts were dried under nitrogen stream and residues were resolved in decane. The analysis was performed using GC-FID (GC17A-AF Vers. 3, Shimadzu Corp., Kyoto, Japan). After extraction of the neutral sterols the samples underwent a strong alkaline hydrolysis with 10 M NaOH solution. The samples were than adjusted to pH 1 with HCl and free bile acids were extracted with diethyl ether. Norcholic acid (Sigma-Aldrich) was added as internal standard and extracts were methylated, silylated and finally dried under nitrogen stream. Residues were resolved in decane and injected in the GC-MS (GC17-QP5000, Shimadzu Corp.). Bile acid quantification was based on the multi-ion detection with m/z = 253.20 for norcholic acid, m/z = 215.25 for iso-lithocholic acid and lithocholic acid, m/z = 73.10 for chenodeoxycholic acid, m/z = 255.3 for hyodeoxycholic acid, and m/z = 458.50 for hyocholic acid.

### RNA isolation and real-time RT-PCR

For the determination of mRNA abundances total RNA was isolated from intestinal mucosa using Trizol™ reagent (Invitrogen, Karlsruhe, Germany) according to the manufacturer’s protocol. The RNA concentration was estimated from the optical density at 260 nm. RNA purity was proofed by agarose gel electrophoresis. A total of 1.2 μg of total RNA was used for cDNA synthesis using the RevertAid™ M-MuLV Reverse transcriptase (Thermo Fisher Scientific Inc., Waltham, MA, USA). For the determination of relative mRNA concentrations real-time detection RT-PCR using the Rotorgene 6000 system (Corbett Research, Mortlake, Australia) was applied. A total of 1 μl cDNA templates were amplified in a total volume of 20 μl using 200 μM dNTPs (Genecraft, Cologne, Germany), 1.5 mM MgCl_2_, 0.5 U GoTaq DNA polymerase, 4 μl 5× buffer (all from Promega, Mannheim, Germany), 0.2 μl 10× SYBR Green (Sigma-Aldrich), and 5.4 pM of each primer. The PCR protocol provided an initial denaturation at 95°C for 3 min and 20–35 cycles of amplification comprising denaturation at 95°C for 25 s, annealing at primer-specific temperatures (57-60°C) for 30 s and elongation at 72°C for 55 s. Subsequently melting curve analysis was performed from 50°C to 99°C with continuous fluorescence measurement. The amplification of a single product of the expected size was confirmed using 2% agarose gel electrophoresis. Relative mRNA concentrations were calculated using the ∆∆C_t_ method [21]. C_t_-values of target genes and the reference genes were obtained using Rotorgene Software 5.0. Succinate dehydrogenase, subunit A (SDHA) and ribosomal protein S9 (RPS9) served as appropriate reference genes. Relative mRNA expression was expressed as fold change of mRNA abundance relative to the casein group. Sequences of the gene-specific primers are shown in Additional file 2.

### Plasma concentrations of 25-hydroxy vitamin D_3_ (25(OH)D_3_) and tocopherols

The plasma concentration of 25(OH)D_3_ was determined by coupled liquid chromatography-mass spectrometry (LC-MS) using a MassChrom™ reagent kit (Chromsystems, Munich, Germany) as described recently [22]. In brief, plasma samples were mixed with deuterated 25(OH)D_3_ (Chemaphor Inc., Ottawa, Canada) as internal standard. Precipitation reagent was added, and subsequent to centrifugation at 15,000 g for 5 min the supernatant was transferred into a HPLC vial and analysed by HPLC (Agilent 1100 HPLC, Agilent Technologies, Waldbronn, Germany), coupled to a MS system (API 2000, Applied Biosystems, Darmstadt, Germany).

The concentration of plasma tocopherol isomers was determined by HPLC analysis [23]. Plasma samples were mixed with 1 ml of a 0.1 g/l pyrogallol solution (ethanol, absolute) and 150 μl saturated NaOH solution. This mixture was incubated at 70°C for 30 min, and tocopherols were extracted with n-hexane. Individual tocopherols of the extracts were separated isocratically by HPLC (Agilent 1100 HPLC, Agilent Technologies) using a mixture of n-hexane and 1,4 dioxane (94:6, v/v) as mobile phase and a LiChrospher Si-60 column (5 μm particle size, 250 mm length, 4 mm internal diameter; Agilent Technologies) and detected by fluorescence (excitation wavelength 295 nm, emission wavelength 325 nm).

### Analysis of plasma and faeces minerals

For the determination of calcium, phosphorus, magnesium and zinc concentrations, plasma was diluted with distilled water and faeces were hydrolysed with 6 M HCl and 1.76 M HNO_3_ according to the official VDLUFA method [12]. The minerals were analysed by inductively coupled plasma optical emission spectroscopy (ICP OES; Varian 715-ES; Agilent Technologies) using selected wavelengths (Ca: 318 nm, P: 215 nm, Mg: 280 nm, Zn: 214 nm). Analyses were run in duplicates.

### Cell culture experiments

Caco-2 cells were cultured in minimal essential medium (MEM) with 10% fetal bovine serum (FBS) at 37°C with 5% CO_2_ and passaged at 90% of confluence [24]. For cholesterol-uptake experiments, cells were seeded in common 6 well plates at a density of 0.8 × 10^6^ cells per well and cultured for 20 to 23 days. Cells were used for experiments from passage 17 to 27. 24 h prior to the experiments cells received serum free medium.

For the cholesterol-uptake, cells were incubated for 2 h at 37°C with 2 mM radioisotopically labelled cholesterol ([4-^14^C]-cholesterol, 0.1 mCi/ml, American Radiolabeled Chemicals Inc., St. Louis, MO, USA) dissolved in MEM plus 2% FBS as previously described [25,26] with or without the following test substances: 0.04 mg/ml ezetimibe (Santa Cruz Biotechnology Inc., Heidelberg, Germany), 5 mg/ml albumin (Sigma-Aldrich), 0.6 mg/ml lupin conglutin γ or 0.18 mg/ml sodium phytate (Sigma-Aldrich). The ezetimibe concentration which we used for the incubation study ranged in the upper concentration levels of ezetimibe used in other cell culture experiments [27,28]. Prior to the incubation, the cells were washed twice with HEPES buffer (25 mM HEPES/Tris (pH 7.5), 140 mM NaCl, 5.4 mM KCl, 1.8 mM CaCl_2_, 0.8 mM MgSO_4_, 5 mM glucose, 37°C). After the incubation, the cells were quickly washed twice with phosphate buffered saline (37°C) containing 1% bovine serum albumin and four times with ice-cold HEPES buffer. Finally, cells were dissolved in radioimmunoprecipitation assay (RIPA) buffer (50 mM Tris/HCl (pH 8.0), 150 mM NaCl, 1% Triton X-100 (w/v), 0.5% sodium deoxycholate (w/v), 0.1% sodium dodecyl sulfate (w/v)) and the amount of absorbed radiolabelled cholesterol was determined by liquid scintillation spectrometry (Liquid Scintillation Analyser Tri-Carb 2800 TR, Perkin Elmer, Rodgau, Germany). Protein concentration was measured using BC assay (Uptima, Interchim, Montlucon, France). Data are expressed as pmol of cholesterol per mg of cell protein.

### Statistical analysis

Values are expressed as means ± SD. Means of the two groups were compared by *Student’s t*-test using the statistic software MINITAB (Release 13, Minitab Ltd., Michigan, USA). Significances of differences between basal and final means were tested by the paired *t*-test. Means were considered significantly different at *P* < 0.05.

## Results

### Daily food intake and final body mass of pigs

Daily food intake (casein group: 1.22 ± 0.04 kg/d; lupin protein group: 1.23 ± 0.03 kg/d) and final body mass (casein group: 49.0 ± 3.0 kg; lupin protein group: 48.8 ± 2.4 kg) did not differ between the two groups of pigs.

### Plasma and liver lipids

**Figure 1:**
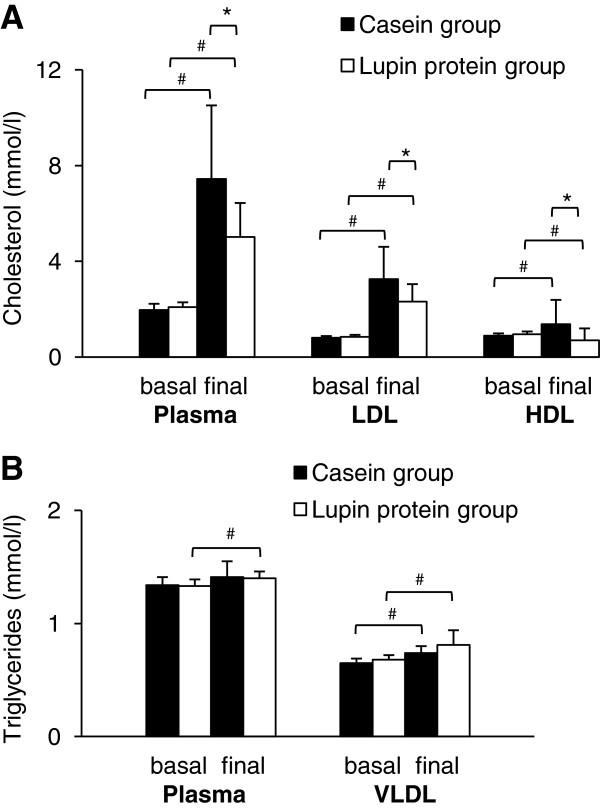
**Cholesterol (A) and triglyceride (B) concentrations in plasma and lipoproteins of pigs fed the experimental diets.** Samples were analysed before (basal) and after (final) feeding diets containing 23% casein or 23% lupin protein isolate for 4 weeks. Values are means (n = 12) with standard deviations. *Significantly different between the two groups at one time point (*Student’s t-*test*, P* < 0.05). ^#^Significantly different between basal and final means within one group (*P* < 0.05, *paired t-*test).

At baseline, the concentration of cholesterol in plasma and lipoproteins was not different between the two groups of pigs (Figure 1A). Pigs treated with the hypercholesterolemic diet for 4 weeks developed significantly higher cholesterol concentrations in plasma, LDL and HDL compared to baseline (Figure 1A; *P* < 0.05), but pigs that were fed the lupin protein isolate had lower final concentrations of plasma, LDL and HDL cholesterol (−32.6%; −28.9% and −16.5%; *P* < 0.05) than pigs fed casein (Figure 1A). The ratios of total to HDL cholesterol (casein group 2.83 ± 0.73 vs. lupin protein group 2.46 ± 0.38) and LDL to HDL cholesterol (casein group 1.15 ± 0.36 vs. lupin protein group 1.13 ± 0.25) did not differ between the two groups. Compared to baseline, the final concentration of triglycerides in plasma increased slightly in the lupin protein group (*P* < 0.05) but not in the casein group. The concentration of VLDL triglycerides increased in both groups of pigs (casein group, *P* < 0.01; lupin protein group, *P* < 0.01), without showing any differences in the final concentrations of VLDL triglycerides between the two groups (Figure 1B). Contents of liver cholesterol (casein group: 9.56 ± 4.10 μmol/g; lupin protein group: 8.50 ± 2.32 μmol/g) and liver triglycerides (casein group: 7.35 ± 0.97 μmol/g; lupin protein group: 6.76 ± 1.62 μmol/g) did not differ between the two groups of pigs.

### Faecal excretion of triglycerides, neutral sterols and bile acids

**Figure 2:**
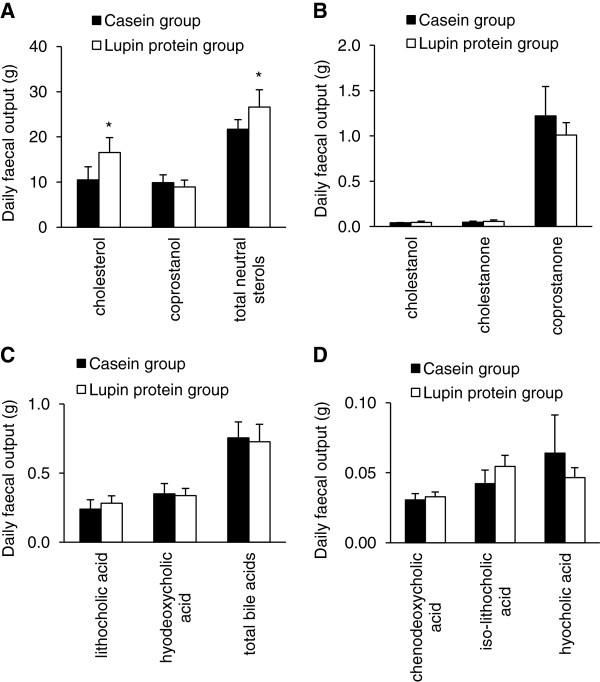
**Daily faecal output of neutral sterols and bile acids of pigs fed the experimental diets.** Pigs received diets containing 23% casein or 23% lupin protein isolate for 4 weeks. **(A)** major neutral sterols cholesterol and coprostanol, and total neutral sterols; **(B)** minor neutral sterols cholestanol, cholestanone and coprostanone; **(C)** major bile acids lithocholic acid and hyodeoxycholic acid, and total bile acids; **(D)** minor bile acids chenodeoxycholic acid, iso- lithocholic acid and hyocholic acid. Faeces were collected quantitatively during the last 5 days of the experiment. Major and minor neutral sterols were analysed by GC-FID, major and minor bile acids were analysed by GC-MS. Values are means (n = 6) with standard deviations. *Significantly different from the casein group (*P* < 0.05, *Student’s t-*test).

Analysis of faeces revealed a higher cholesterol excretion in pigs that were fed the lupin protein isolate than in pigs receiving casein (+ 57.1%; *P* < 0.05; Figure 2A). The faecal excretion of coprostanol, cholestanol, cholestanone, coprostanone, and bile acid derivatives did not differ between the two groups of pigs (Figure 2A,B,C and D). Lupin protein isolate also did not influence the faecal output of triglycerides compared to casein (casein group: 0.68 ± 0.27 g/d; lupin protein group: 0.65 ± 0.44 g/d).

### Relative mRNA concentrations of intestinal sterol transporters and receptors

**Figure 3:**
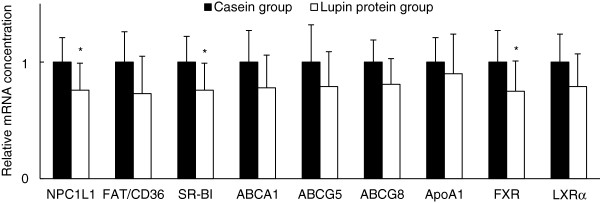
**Relative mRNA concentrations of sterol transporters and receptors in intestinal mucosa of pigs fed the experimental diets.** Pigs received diets containing 23% casein or 23% lupin protein isolate for 4 weeks. Niemann-Pick C1-like 1 (NPC1L1), fatty acid translocase/Cluster determinant 36 (FAT/CD36), scavenger receptor class B, type 1 (SR-BI), ATP-binding cassette transporters (ABC) A1, G5, and G8, apolipoprotein A-I (ApoA1), farnesoid X receptor (FXR), and liver X receptor (LXR) α. Values are means (n = 12) with standard deviations. The mRNA concentrations of succinate dehydrogenase, subunit A (SDHA) and ribosomal protein S9 (RPS9) were used for normalization. The mRNA concentrations of the genes are shown relative to those of pigs fed the casein diet (= 1.00). *Significantly different from the casein group (*P* < 0.05, *Student’s t-*test*)*.

Relative mRNA abundances of the intestinal transporters Niemann-Pick C1-like 1 (NPC1L1) and scavenger receptor class B, type 1 (SR-BI), which are involved in the transport of cholesterol from the intestinal lumen into the intestinal epithelial cells, were significantly lower in the lupin protein isolate fed pigs than in the casein fed pigs (*P* < 0.05, Figure 3). The relative mRNA concentrations of fatty acid translocase/Cluster determinant 36 (FAT/CD36), which is involved in cholesterol absorption, the ATP-binding cassette transporter (ABC) G8 which catalyses the reverse cholesterol transport and the basolateral cholesterol transporter ABCA1 tended to be lower in the lupin protein group than in the casein group (*P* = 0.05, *P* = 0.06 and *P* = 0.08, respectively; Figure 3). The relative mRNA concentration of ABCG5 did not differ significantly between the two treatment groups (Figure 3). The relative mRNA concentration of the farnesoid X receptor (FXR), a transcription factor which is activated by bile acids was significantly lower in the lupin protein group compared to the casein group (*P* < 0.05, Figure 3). Pigs from the lupin protein group tended to express less mRNA of the liver X receptor alpha (LXRα) (*P* = 0.09; Figure 3) compared to the casein fed animals. There was no difference in ApoA1 mRNA expression between the two groups.

### Plasma 25-hydroxy vitamin D_3_ and plasma tocopherol

**Figure 4:**
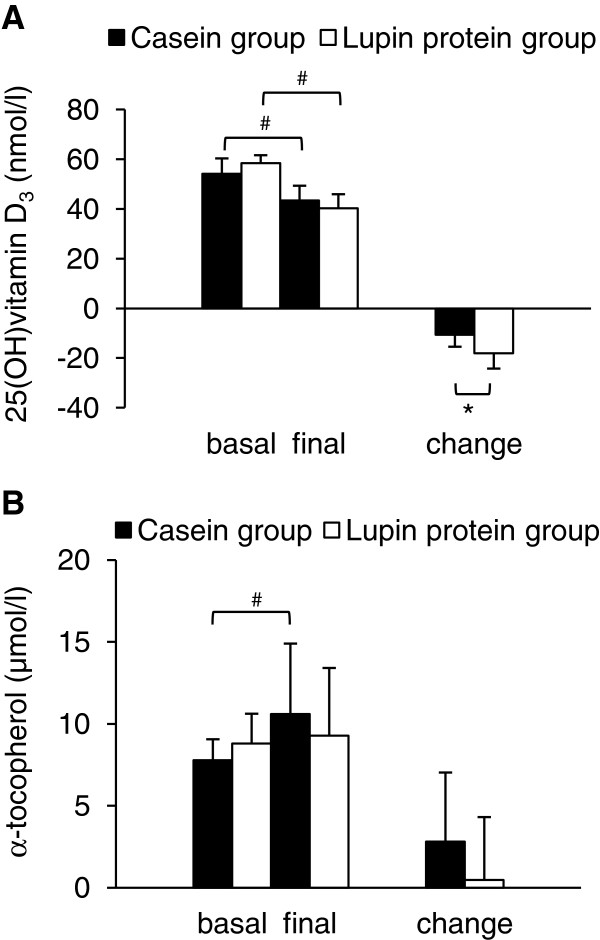
**Plasma concentrations of 25-hydroxy vitamin D**_**3 **_**(A) and α-tocopherol (B) of pigs fed experimental diets.** Samples were analysed before (basal) and after (final) feeding diets containing 23% casein or 23% lupin protein isolate for 4 weeks. Values are means (n = 12) with standard deviations. *Significantly different between the two groups (*P* < 0.05, *Student’s t-*test*)*. ^#^Significantly different between basal and final means within one group (*P* < 0.05, paired *t*-test).

Plasma concentrations of 25(OH)D_3_ at the beginning and at the end of the experiment did not differ between the two groups of pigs. Considering the changes of plasma 25(OH)D_3_ within the experimental period, lupin protein isolate fed pigs revealed a stronger reduction of the plasma concentration than casein fed pigs (Figure 4A). Tocopherol isomer analysis revealed no detectable concentrations of the tocopherols β, γ and δ in plasma. In pigs fed casein, final plasma concentration of tocopherol α was higher than at the beginning of the experiment (*P* < 0.05), whereas such an increase of tocopherol α concentration within the experimental period was not observed in lupin protein isolate fed pigs (Figure 4B). Comparing the changes of plasma tocopherol α concentration within the 4 weeks of treatment, increase of circulating tocopherol α tended to be lower in the lupin protein group than in the casein group (Figure 4B).

### Plasma mineral concentrations and faecal output of minerals

**Figure 5:**
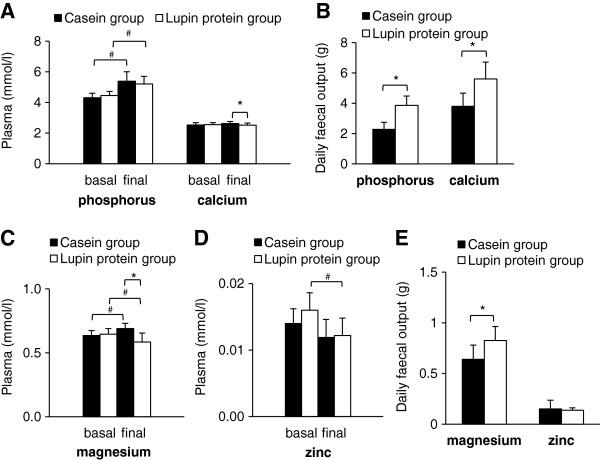
**Plasma mineral concentrations and daily faecal mineral output of pigs fed experimental diets.** Plasma samples were analysed before (basal) and after (final) feeding diets containing 23% casein or 23% lupin protein isolate for 4 weeks. Plasma concentrations of phosphorus and calcium **(A)**, magnesium **(C)** and zinc **(D)** and daily faecal output of phosphorus and calcium **(B)**, magnesium and zinc **(E)** were determined. Faeces were collected quantitatively during the last 5 days of the experiment. Values are means (plasma, n = 12; faecal output, n = 6), with standard deviations. *Significantly different between the two groups at one time point (*P* < 0.05, *Student’s t*-test*)*. ^#^Significantly different between basal and final means within one group (*P* < 0.05, paired *t*-test).

Basal plasma concentrations of total phosphorus, calcium, magnesium and zinc did not differ between the two groups of pigs (Figure 5A,C and D). In both treatment groups, circulating concentrations of total phosphorus increased within the 4 weeks of the experimental period, indicating raised concentrations of circulating phospholipids in response to the hypercholesterolemic diet. Final concentrations of total phosphorus did not differ between the two groups of pigs, whereas final plasma concentrations of calcium and magnesium were lower in the group that received the lupin protein diet than in the group fed the casein diet (Figure 5A and C). Pigs fed the lupin protein isolate revealed lower plasma concentrations of zinc compared to baseline (*P* < 0.05, Figure 5D). Baseline and final concentrations of plasma zinc did not differ in pigs that received the casein diet. Faecal output revealed a higher excretion of phosphorus, calcium and magnesium in pigs fed lupin protein isolate than in pigs fed casein (Figure 5B and E). Faecal output of zinc did not differ between the two treatment groups (Figure 5E).

### Cholesterol-uptake by Caco-2 cells

**Figure 6:**
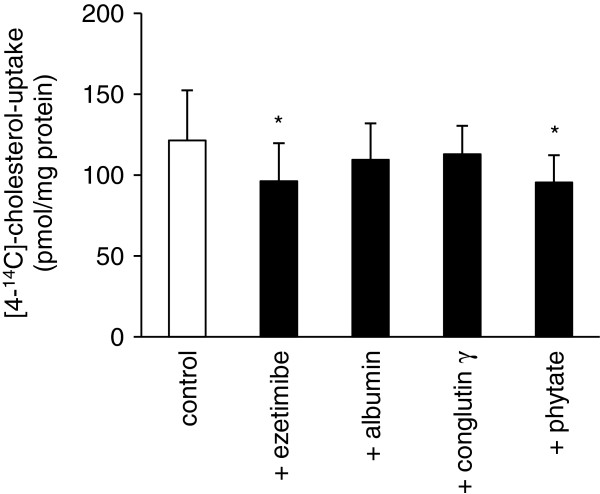
**[4-**^**14**^**C]-cholesterol-uptake by Caco-2 cells during a 2 h-incubation with ezetimibe, albumin, conglutin γ or phytate.** Cells were cultured for 20–23 days before the uptake experiments. MEM + 2% bovine serum albumin without supplements served as control medium. Concentrations of test substances: 0.04 mg/ml ezetimibe, 5 mg/ml albumin, 0.06 mg/ml conglutin γ and 0.18 mg/ml phytate. Values are means (n = 3–5), with standard deviations. *Significantly different from control (*P* < 0.05, *Student’s t*-test*)*.

Caco-2 cells treated with the cholesterol-uptake inhibitor ezetimibe revealed a 20.5% lower uptake of [4-^14^C]-cholesterol than the control cells (*P* < 0.05, Figure 6), confirming the functioning of the cell model. The amount of cholesterol taken up by the cells was also decreased in cells that were treated with phytate (−21.1%, *P* < 0.05; Figure 6). Neither conglutin γ nor albumin, which served as control protein, revealed any influence on cholesterol-uptake compared to the control.

## Discussion

The current study with pigs as a model for human gastrointestinal and lipid physiology reveals hypocholesterolemic effects of lupin protein isolate compared to casein, and confirms previous findings from lupin protein studies with hypercholesterolemic rats and humans [2,4]. We found that lupin protein fed pigs had lower levels of both, the undesirable LDL cholesterol and the favourable HDL cholesterol, compared to pigs that received casein, which in turn resulted in comparable ratios of total to HDL cholesterol and LDL to HDL cholesterol in both groups of pigs. Considering the fact that those ratios are much better predictors of risk of heart diseases than LDL or total cholesterol levels alone, we assume that the cholesterol-lowering properties of lupin protein isolate may not be inevitably associated with a risk reduction of cardiovascular diseases. Faeces analyses from this study showed that the reduction of circulating plasma cholesterol concentrations in response to lupin protein isolate administration was associated with a significant increase of faecal cholesterol output. We therefore assume that the increased faecal cholesterol excretion in the lupin protein group compared to the casein group was –at least partially– responsible for the reduced concentrations of circulating plasma, LDL and HDL cholesterol in these pigs. As there are no differences between the two groups in the relative mRNA abundance of ApoA1, which is a major protein component of HDL, we assume that the intestinal ApoA1 expression is not the leading cause for the observed HDL lowering effects. Data further show that the faecal output of bile acids, and also coprostanol, cholestanol, coprostanone, and cholestanone, which normally arise from the conversion of cholesterol in the gut by intestinal bacteria [29], was not altered in response to the treatment with lupin protein isolate. In this study we used extremely high amounts of dietary cholesterol (2%), we may therefore assume that the capacity of coprostanoligenic bacteria to convert cholesterol to coprostanol was not high enough for further increase of the faecal coprostanol content in the lupin protein fed group.

Different factors could have caused the increased output of faecal cholesterol in the lupin protein group: (1) lupin protein isolate compared to casein could have affected the cholesterol absorption by modifying sterol transporter expression in the intestine, (2) lupin protein isolate could have un-specifically bound sterols in the gut lumen which in consequence increased cholesterol output, (3) lupin protein isolate could have contributed to an increased excretion of cholesterol via the bile, (4) lupin protein isolate could have influenced formation, solubility or composition of micelles which in turn affected cholesterol-uptake into the enterocytes or (5) lupin protein isolate could have affected the cholesterol efflux via modification of basolateral sterol transporter expression in the intestine. Several intestinal transporters are involved in trafficking cholesterol across the apical enterocyte membrane such as SR-BI [30], FAT/CD36 [31], NPC1L1 [32] and ABC transporters [33]. All of these transporters have been found to be regulated by transcription [34]. Here we show that lupin protein isolate, compared to casein, down-regulated the mRNA abundances of intestinal sterol transporters, especially those which catalyse the cholesterol-uptake. This is further supported by the fact that lupin protein fed pigs tended to have lower mRNA expression of the cholesterol responsive nuclear receptor LXRα and its responsive gene ABCA1 which are indicative of reduced intracellular cholesterol in the intestinal cells of these animals. Similar effects on LXRα and ABCA1 expression were also observed in hyperinsulinemic obese Zucker rats that were fed with soy protein [35]. Thus, the mRNA data of the current experiment are indicative of a lower intestinal cholesterol absorption following intake of lupin protein isolate, and not of an altered cholesterol efflux.

There is a growing body of evidence that certain proteins such as those from buckwheat seeds [8], egg white [36], silk protein sericin [37] and soystatin, a novel peptide derived from soybean protein [38], may influence the incorporation of cholesterol into micelles. Nagaoka et al. could show that the soybean protein derived Val-Ala-Trp-Trp-Met-Tyr peptide, designated soystatin, inhibits cholesterol absorption by reducing the micellar solubility of cholesterol [38]. Lupin is genetically similar to soybean and one can assume that the diminished solubilisation of cholesterol in micelles may also occur in response to ingested lupin proteins. Intriguingly, soystatin arises from glycinin, the soybean 11S globulin, and the lupin homologous protein [UniProt:Q53I54] does contain a resembling sequence, namely Ile-Pro-Phe-Trp-Met-Tyr between amino acids 152 and 157. Polar lipids such as fatty acids are normally well absorbed from emulsions, and incorporation of polar lipids into mixed micelles is not necessarily a prerequisite for their absorption [39]. However, the formation of micelles is essential for the absorption of cholesterol and fat-soluble vitamins, because those non-polar lipids, which position themselves between the oil and the micellar phase, must be carried by micelles to the brush border membrane [40]. Analyses of the plasma concentrations of 25(OH)D_3_ and tocopherols indicate negative impacts of lupin protein isolate on micellar transport system because changes of plasma concentrations of both vitamins in response to lupin protein isolate revealed a trend toward lower values compared to casein. On the other hand there are particles in the plasma, like LDL, which contain tocopherols. Therefore, the decreased LDL cholesterol concentrations of pigs fed lupin protein isolate compared to those fed casein could also explain the lower plasma α-tocopherol concentrations.

Another biofunctional molecule which is naturally associated with the lupin protein isolate is phytic acid. Phytic acid is suggested to possess also hypocholesterolemic properties. Lee and co-workers found that diabetic KK mice that were fed diets supplemented with 0.5, 1.0, and 1.5% of phytic acid developed a dose-dependent reduction of plasma cholesterol concentrations that was mainly caused by LDL reduction [41]. Phytic acid also developed plasma cholesterol-lowering activity in tilapia [42] and rats fed a high-sucrose diet [43], and increased faecal cholesterol and bile acid excretion in rats and mice [44,45]. Koba et al. [46] investigated the influence of phytate on the cholesterol-lowering effect of soy protein. Thereby, rats fed a cholesterol-enriched diet with soy protein and phytate-replenished soy protein showed significantly lower serum and liver cholesterol concentrations than rats fed casein and phytate-depleted soy protein. Analysis of the lupin protein isolate which we used for the pig study contained 25 g/kg phytic acid which corresponds to an additional content of 0.57% phytic acid in the lupin protein diet. Seeds of legumes naturally contain relatively high amounts of phytic acid [47-49], and food technology processes which are used to isolate proteins from plants normally do not separate phytic acid from the proteins. Phytate serves as storage of phosphorus for the plant kernels and has been regarded as an antinutrient because it inhibits the absorption of essential trace elements and minerals (for review see Schlemmer et al. [50]). However, growing evidence indicates that phytic acid exerts also beneficial effects such as antioxidative functions [51,52], and antitumor activity [53]. Data from our *in vitro* experiment show that phytate was capable of reducing the cellular cholesterol-uptake to a similar magnitude as ezetimibe, a known cholesterol absorption inhibitor [54], whereas conglutin γ was non-effective. Thus, we assume that phytate associated with the lupin protein isolate could have contributed to the observed cholesterol-lowering effect in the group fed the lupin protein diet. However, the increased faecal output of calcium and magnesium and the lower plasma concentrations of both minerals, therefore, constitute an undesirable side effect of the cholesterol-lowering activity of lupin protein isolate-associated phytate.

## Conclusions

Collectively, the current data show that lupin protein isolate compared to casein stimulates faecal output of cholesterol which is suggested to be responsible for the cholesterol-lowering effect of lupin proteins. The findings that lupin protein isolate treated pigs had reduced relative mRNA concentrations of intestinal NPC1L1 and SR-BI and lower plasma concentrations of fat-soluble vitamins support the assumption that lupin proteins negatively affect intestinal cholesterol absorption. The cholesterol-uptake data provide evidence that associated phytate could – at least partly – contribute to the observed cholesterol-lowering effect of lupin protein isolate.

## Abbreviations

ABC: ATP-binding cassette transporter; ApoA1: apolipoprotein A-I; CYP7A1: Cholesterol 7α-hydroxylase; DM: Dry matter; FAT/CD36: Fatty acid translocase/cluster determinant 36; FBS: Fetal bovine serum; FID: Flame ionization detector; FXR: Farnesoid X receptor; LXRα: Liver X receptor α; HDL: High density lipoprotein; ICP-OES: Inductively coupled plasma optical emission spectroscopy; LC: Liquid chromatography; LDL: Low density lipoprotein; MEM: Minimum essential medium; MS: Mass spectrometry; NPC1L1: Niemann-Pick C1-like 1; RIPA: Radioimmunoprecipitation assay; RPS9: Ribosomal protein S9; SDHA: Succinate dehydrogenase, subunit A; SR-BI: Scavenger receptor class B, type 1; VLDL: Very low density lipoprotein; 25(OH)D3: 25-hydroxy vitamin D_3_.

## Competing interests

The authors declare that they have no competing interests.

## Authors’ contributions

GIS supervised the study and wrote the manuscript with assistance from all other authors, especially JR and AS. JR and HK carried out the animal experiment, JR and AS analysed faecal minerals and mRNA data, CB separated plasma lipoproteins, SK, GJ and JR analysed neutral sterols and bile acids in faeces, FH analysed 25(OH)D_3_ and α-tocopherol in plasma samples. MMD provided the isolated conglutin γ fraction. JR and SG performed cholesterol-uptake experiments with Caco-2 cells. All authors read and approved the final version of the manuscript.

## Supplementary Material

Additional file 1: Table S1 Amino acid composition of experimental proteins. The table contains the amino acid concentration (g/kg) of the experimental proteins casein and lupin protein isolate. Click here for file

Additional file 2: Table S2 Primer sequences used in real-time RT-PCR. The table lists the sequences of the used primers and the accession numbers of the analysed genes. Click here for file
