# Supplementary material for: Lupin protein isolate versus casein modifies cholesterol excretion and mRNA expression of intestinal sterol transporters in a pig model
Source: Nutr Metab (Lond). 2014 Feb 3;11:9. doi: 10.1186/1743-7075-11-9 (PMC3922606; doi:10.1186/1743-7075-11-9)
Supplement: Additional file 1: Table S1 — Amino acid composition of experimental proteins. The table contains the amino acid concentration (g/kg) of the experimental proteins casein and lupin protein isolate. [file 1743-7075-11-9-S1.pdf]

Additional file 1

Table 1 Amino acid composition of experimental proteins

**Amino acids**	**Casein (g/kg)**	**Lupin protein isolate (g/kg)**
Alanine	23.6	25.0
Arginine	28.7	92.2
Aspartic acid + Asparagine	56.3	86.4
Cysteine	2.5	9.8
Glutamic acid + Gutamine	175.3	186.6
Glycine	14.4	32.1
Histidine	23.0	20.0
Isoleucine	40.9	36.6
Leucine	76.2	63.4
Lysine	63.9	33.6
Methionine	23.0	2.9
Phenylalanine	41.7	35.5
Proline	90.6	35.8
Serine	44.7	39.9
Threonine	33.5	25.6
Tyrosine	36.4	25.2
Tryptophane	11.1	7.5
Valine	51.1	31.1
