# Supplementary material for: Lupin protein isolate versus casein modifies cholesterol excretion and mRNA expression of intestinal sterol transporters in a pig model
Source: Nutr Metab (Lond). 2014 Feb 3;11:9. doi: 10.1186/1743-7075-11-9 (PMC3922606; doi:10.1186/1743-7075-11-9)
Supplement: Additional file 2: Table S2 — Primer sequences used in real-time RT-PCR. The table lists the sequences of the used primers and the accession numbers of the analysed genes. [file 1743-7075-11-9-S2.pdf]

Additional file 2

Table 2 Primer sequences used in real-time RT-PCR

Gene	Forward primer	Reverse primer	Accession number
ABCA1	CGGAGAAAGAAGTTGGCAAG	TCGGTTCATCCAGAAAGACC	XM_003480560
ABCG5	CCTGTCCAGGTGCAACATCCAGAT	TCAAGGCCAGCATGGATCCAAGG	EF472591.1
ABCG8	GAGCTTCAGAGTGAGGAGCGGACA	GAAGGTTCTGGGCAGGCGCA	EF472592.1
ApoA1	CGATCAAAGACAGTGGCAGA	GCTGCACCTTCTTCTTCACC	NM_214398.1
FAT/CD36	ATCGTGCCTATCCTCTGG	CCAGGCCAAGGAGGTTAA	NM_001044622.1
FXR	TATGAACTCAGGCGAATGCCTGCT	ATCCAGATGCTCTGTCTCCGCAAA	KF597010.1
LXRα	AGAACAGATCCGCCTGAAGA	GGTCTGAAAAGGAGCGTCTG	AB254405.1
NPC1L1	TGTCCCCGCCTCTACACCGG	GGCCACGCGAGTCACGTTGA	XM_003134893.1
RPS9	GTCGCAAGACTTATGTGACC	AGCTTAAAGACCTGGGTCTG	XM_005664825
SDHA	CTACGCCCCCGTCGCAAAGG	AGTTTGCCCCCAGGCGGTTG	XM_005659031
SR-BI	TGGCTCCCAACACCTTATTC	ACACTGCCTCTGCCAGAACT	NM_213967.1

ABCA1, ATP-binding cassette transporter A1; ABCG5, ATP-binding cassette transporter G5; ABCG8, ATP-binding cassette transporter G8; ApoA1, apolipoprotein A-I; FAT/CD36, Fatty acid translocase/Cluster determinant 36; FXR, farnesoid X receptor, LXRα, liver X receptor α; NPC1L1, Niemann-Pick C1-like 1; RP S9, ribosomal protein S9; SDHA, succinate dehydrogenase, subunit A; SR-BI, scavenger receptor class B, type 1.
